# Supplementary material for: Intensive community care services for adolescents with acute psychiatric emergencies: trial feasibility findings from the pilot phase of a multi-centre randomised controlled trial
Source: BMC Psychiatry. 2026 Feb 19;26:247. doi: 10.1186/s12888-025-07528-2 (PMC13001206; doi:10.1186/s12888-025-07528-2)
Supplement: Supplementary file 1 — Supplementary Material 1 [file 12888_2025_7528_MOESM1_ESM.docx]

**Intensive Community Care Services for Adolescents with Acute Psychiatric Emergencies: Trial Feasibility Findings from the Pilot Phase of a Multi-centre Randomised Controlled Trial**

Dennis Ougrin^1^, Thilipan Thaventhiran^1^, Ben Hoi-Ching Wong^2^, Izabela Pilecka^3^, Sabine Landau^3^, Sarah Byford^3^, Petrina Chu^3^, Hassan Jafari^3^, Margaret Heslin^3^, Emma Tassie^3^, Paula Reavey^4^, Toby Zundel^5^, Mandy Wait^5^, Ruth Woolhouse^2^, Tauseef Mehdi^6^, Jovanka Tolmac^7^, Joe Clacey^8^, Leon Wehncke^9^, Veronika Beatrice Dobler^10^, Rhys Bevan-Jones^11^

^1^Wolfson Institute of Population Health, Queen Mary University of London, London, UK; ^2^East London NHS Foundation Trust, London, UK; ^3^King’s College London, London, UK; ^4^London South Bank University, London, UK; ^5^South London and Maudsley NHS Foundation Trust, Beckenham, UK; ^6^Berkshire Healthcare NHS Foundation Trust, Bracknell, UK; ^7^Central and North-West London NHS Foundation Trust, London, UK; ^8^Oxford Health NHS Foundation Trust, Oxford, UK; ^9^North-East London NHS Foundation Trust, London, UK; ^10^Cambridgeshire and Peterborough Foundation Trust, Cambridge, UK; ^11^Cardiff University, Wales, UK.

**SUPPLEMENTARY MATERIALS**

**Supplementary Appendix 1.**

**Table S1. Intervention and control costs and sources**

| **Service** | **Unit cost or range** | **Source** | **Notes** |
| --- | --- | --- | --- |
| Psychiatrist ICCS worker (per contact) | 367.07 | NHS Reference Costs 2021-22 | Child and Adolescent Psychiatry Service: Non-Admitted Non-Face-to-Face Attendance, Follow-up. |
| Psychiatrist non-ICCS worker (per contact) | 367.07 | NHS Reference Costs 2021-22 | Child and Adolescent Psychiatry Service: Non-Admitted Non-Face-to-Face Attendance, Follow-up. |
| Qualified CAMHS worker with ICCS worker (per contact) | 54.33 | Jones et al., 2022 | Average of Band 5 and Band 6, assuming 1 hour. |
| Qualified CAMHS worker with non-ICCS worker (per contact) | 54.33 | Jones et al., 2022 | Average of Band 5 and Band 6, assuming 1 hour. |
| Unqualified CAMHS worker with ICCS worker (per contact) | 37 | Jones et al., 2022 | Assuming 1 hour Band 4. |
| Unqualified CAMHS worker with non-ICCS worker (per contact) | 37 | Jones et al., 2022 | Assuming 1 hour Band 4. |
| Full days on day patient ward (per day) | 412.92 | NHS Reference Costs 2021-22 | Children and Adolescent Mental Health Services, Day Care Facilities. |
| Half days on day patient ward (per half day) | 206.46 | NHS Reference Costs 2021-22 | Children and Adolescent Mental Health Services, Day Care Facilities. |
| Admitted nights to psychiatric inpatient services (per night) | 302.74 to 1,450.33 | NHS Reference Costs 2017-18 | Dependent on type of ward. Inflated from 2017/18. |

**Table S2. Service use unit costs and sources**

| **Service** | **Unit cost** | **Source** | **Notes** |
| --- | --- | --- | --- |
| **Hospital** | | | |
| Inpatient for mental health reason (per night) | 782.55 | NHS Reference Costs 2017-18 | Inflated from 2017/18. |
| Inpatient for physical health reason (per night) | 885.45 | NHS Reference Costs 2017-18 | Inflated from 2017/18. |
| Day case for mental health reason (per appointment) | 412.92 | NHS Reference Costs 2017-18 | Children and adolescent mental health services, day care facilities, mental health tab. |
| Day case for physical health reason (per appointment) | 772.06 | NHS Reference Costs 2021-22 | Weighted average of all paediatric contacts cost per day case. |
| Outpatient for mental health reason (per appointment) | 383.46 | NHS Reference Costs 2021-22 | Children and adolescent mental health services, outpatient attendances, mental health tab. |
| Outpatient for physical health reason (per appointment) | 263.29 | NHS Reference Costs 2021-22 | Weighted average paediatric consultant-led outpatient attendance; paediatric non-consultant led outpatient attendance. |
| A&E (per attendance) | 144.00 | NHS Reference Costs 2021-22 | Total outpatient attendance tab, emergency medicine service code 180. |
| Ambulance (care or transport) (per attendance) | 347.50 | NHS Reference Costs 2021-22 | Weighted average of see and treat and see and convey in ambulance tab. |
| Health based place of safety | 1,236.00 | Heslin et al., 2015 | Inflated from 2013/14 |
| **Community (per contact)** | | | |
| GP | 46.16 | Jones et al., 2022 | Average of 9.22 minute GP surgery consultation, cost of telephone triage and cost of home visit. |
| Practice nurse | 11.62 | Jones et al., 2022 | Assuming 15.5 minute appointment. |
| Any other nurse | 16.53 | Jones et al., 2022 | Average of Band 5 and Band 6, assuming 15.5 minute appointment. |
| CAMHS worker | 55 | Jones et al., 2022 | Band 6 community-based scientific and professional staff per hour. |
| Any therapist providing talking therapy in the community | 165 | Jones et al., 2022 | Average of Clinical psychologist service and other therapist adult one to one NHS reference costs. |
| Psychiatrist in the community | 367.07 | NHS Reference Costs 2021-22 | Child and Adolescent Psychiatry Service: Non-Admitted Non-Face-to-Face Attendance, Follow-up. |
| Crisis resolution team / home treatment team worker | 437.39 | NHS Reference Costs 2021-22 | Children and Adolescent Mental Health Services, Community Contacts, Crisis Resolution Home Treatment, MH tab. |
| Educational mental health practitioner | 82.18 | Jones & Burns, 2021 | School-based children’s health core (other) services – one to one. Inflated from 2021. |
| Community paediatrician | 350.00 | NHS Reference Costs 2021-22 | Community Paediatric Service, 290. |
| Special educational needs coordinator | 18.80 | Jones et al., 2022 | Assuming 30 minute appointment, 0.61 per minute. |
| Social worker, social services youth worker or family support worker | 47.25 | Jones & Burns, 2021 | Assuming 1 hour social worker (children’s services) appointment. Inflated from 2021. |
| Youth offending team worker | 23.29 | Jones & Burns, 2021 | Assuming 30 minute social worker (children’s services) appointment. Inflated from 2021. |
| Occupational therapist | 42.00 | Jones et al., 2022 | Assuming 1 hour Band 5 appointment. |
| Art/drama/music therapist | 66.00 | Jones et al., 2022 | Community services, average cost per group session (one-to-one) |
| Speech and language therapist | 42.00 | Jones et al., 2022 | Assuming 1 hour Band 5 appointment. |
| Accommodation key worker | 23.63 | Jones & Burns, 2021 | Assuming 30 minute social worker (children’s services) appointment. Inflated from 2021. |
| Drug/alcohol support worker | 40.00 | Jones et al., 2022 | Assuming 30 minute appointment with Alcohol health worker/Alcohol liaison nurse/Substance misuse nurse. |
| Any helpline or advice service | 4.00 | Nspcc.org.uk |  |
| Community nurse | 29.00 | Jones et al., 2022 | Assuming 30 minute Band 6 nurse appointment. |
| NHS psychiatrist | 367.07 | NHS Reference Costs 2021-22 | Child and Adolescent Psychiatry Service: Non-Admitted Non-Face-to-Face Attendance, Follow-up. |
| Health in Mind or the Psychological Wellbeing Service | 55.00 | Jones et al., 2022 | Assuming 1 hour counsellor Band 6 community-based scientific and professional staff appointment. |
| Any other NHS therapist for mental health support | 55.00 | Jones et al., 2022 | Assuming 1 hour for Band 6 community-based scientific and professional staff appointment. |

**Table S3.** **Mental Health Contacts (MHC) between participants and mental health workers over the 6-month observation period**

| **MHC data availability** | **TAU (N=21)** | **ICCS (N=15)** | **Overall (N=36)** |
| --- | --- | --- | --- |
| MHC data available | 16 (76.2%) | 15 (100.0%) | 31 (86.1%) |
| Missing all MHC data | 5 (23.8%) | 0 (0.0%) | 5 (13.9%) |
| **For those with MHC data** | | | |
| Contacts received from psychiatrist ICCS workers | | | |
| Median (IQR) | 0.0 (0.0-0.5) | 0.0 (0.0-5.0) | 0.0 (0.0-3.0) |
| Range | 0.0 - 16.0 | 0.0 - 10.0 | 0.0 - 16.0 |
| Contacts received from psychiatrist non-ICCS workers | | | |
| Median (IQR) | 0.0 (0.0-2.5) | 0.0 (0.0-0.0) | 0.0 (0.0-1.0) |
| Range | 0.0 - 13.0 | 0.0 - 2.0 | 0.0 - 13.0 |
| Contacts received from qualified CAMHS ICCS workers | | | |
| Median (IQR) | 0.0 (0.0-0.0) | 11.0 (5.0-15.0) | 1.0 (0.0-11.0) |
| Range | 0.0 - 17.0 | 0.0 - 22.0 | 0.0 - 22.0 |
| Contacts received from qualified CAMHS non-ICCS workers | | | |
| Median (IQR) | 2.5 (1.5-5.5) | 5.0 (1.0-7.0) | 3.0 (1.0-6.0) |
| Range | 0.0 - 10.0 | 0.0 - 30.0 | 0.0 - 30.0 |
| Contacts received from unqualified CAMHS ICCS workers | | | |
| Median (IQR) | 0.0 (0.0-0.0) | 0.0 (0.0-1.0) | 0.0 (0.0-0.0) |
| Range | 0.0 - 0.0 | 0.0 - 5.0 | 0.0 - 5.0 |
| Contacts received from unqualified CAMHS non-ICCS workers | | | |
| Median (IQR) | 0.0 (0.0-0.0) | 0.0 (0.0-0.0) | 0.0 (0.0-0.0) |
| Range | 0.0 - 7.0 | 0.0 - 0.0 | 0.0 - 7.0 |
| Total days as day patient | | | |
| Mean (SD) | 1.1 (4.5) | 2.7 (10.6) | 1.9 (7.9) |
| Range | 0.0 - 18.0 | 0.0 - 41.0 | 0.0 - 41.0 |

**Table S4. Treatment exposure data over the 6-month observation period**

| **Treatment exposure data availability** | **TAU**  **(N=21)** | **ICCS**  **(N=15)** | **Overall**  **(N=36)** |
| --- | --- | --- | --- |
| Treatment exposure data available | 16 (76.2%) | 15 (100.0%) | 31 (86.1%) |
| Missing all treatment exposure data | 5 (23.8%) | 0 (0.0%) | 5 (13.9%) |
| **For those with treatment exposure data** | | | |
| ICCS contacts offered | | | |
| Median (IQR) | 0.0 (0.0-0.0) | 14.0 (6.0- 8.0) | 1.0 (0.0-14.0) |
| Range | 0.0 - 28.0 | 0.0 - 26.0 | 0.0 - 28.0 |
| ICCS contacts received | | | |
| Median (IQR) | 0.0 (0.0-0.0) | 11.0 (6.0-16.0) | 1.0 (0.0-12.0) |
| Range | 0.0 - 20.0 | 0.0 - 23.0 | 0.0 - 23.0 |
| CAMHS contacts offered by ICCS | | | |
| Median (IQR) | 0.0 (0.0-0.0) | 0.0 (0.0-0.0) | 0.0 (0.0-0.0) |
| Range | 0.0 - 0.0 | 0.0 - 41.0 | 0.0 - 41.0 |
| CAMHS contacts received from ICCS | | | |
| Median (IQR) | 0.0 (0.0-0.0) | 0.0 (0.0-0.0) | 0.0 (0.0-0.0) |
| Range | 0.0 - 0.0 | 0.0 - 38.0 | 0.0 - 38.0 |
| CAMHS contacts offered by non-ICCS teams | | | |
| Median (IQR) | 0.0 (0.0-0.0) | 0.0 (0.0-0.0) | 0.0 (0.0-0.0) |
| Range | 0.0 - 26.0 | 0.0 - 7.0 | 0.0 - 26.0 |
| CAMHS contacts received from non-ICCS teams | | | |
| Median (IQR) | 0.0 (0.0-0.0) | 0.0 (0.0-0.0) | 0.0 (0.0-0.0) |
| Range | 0.0 - 18.0 | 0.0 - 6.0 | 0.0 - 18.0 |
| Standard community contacts offered | | | |
| Median (IQR) | 8.0 (2.5-15.0) | 5.0 (1.0-13.0) | 7.0 (2.0-14.0) |
| Range | 0.0 - 22.0 | 0.0 - 15.0 | 0.0 - 22.0 |
| Standard community contacts received | | | |
| Median (IQR) | 5.5 (2.5-9.5) | 5.0 (1.0-8.0) | 5.0 (2.0-9.0) |
| Range | 0.0 - 16.0 | 0.0 - 14.0 | 0.0 - 16.0 |
| National/Specialist contacts offered | | | |
| Median (IQR) | 0.0 (0.0-0.0) | 0.0 (0.0-0.0) | 0.0 (0.0-0.0) |
| Range | 0.0 - 1.0 | 0.0 - 20.0 | 0.0 - 20.0 |
| National/Specialist contacts received | | | |
| Median (IQR) | 0.0 (0.0-0.0) | 0.0 (0.0-0.0) | 0.0 (0.0-0.0) |
| Range | 0.0 - 1.0 | 0.0 - 18.0 | 0.0 - 18.0 |
| Total number psychological interventions received | | | |
| Median (IQR) | 0.0 (0.0-1.0) | 2.0 (0.0-16.0) | 0.0 (0.0-7.0) |
| Range | 0.0 - 15.0 | 0.0 - 24.0 | 0.0 - 24.0 |

**Table S5. Use of health and social care services in the three months before baseline**

Abbreviations: MH Mental Health; PH Physical Health; A&E Accident and Emergency; GP General Practitioner; CAMHS Child and Adolescent Mental Health Service; CRT Crisis Resolution Team, HTT Home Treatment Team.

|  | **All (n=28)** | | | **ICCS (n=14)** | | | **TAU (n=14)** | | |
| --- | --- | --- | --- | --- | --- | --- | --- | --- | --- |
| **Service** | **Mean (SD)** | **Range** | **% using** | **Mean (SD)** | **Range** | **% using** | **Mean (SD)** | **Range** | **% using** |
| **Hospital** | | | | | | | | | |
| MH inpatient  Nights | 11.29 (22.78) | 0-91 | 78.57 | 7.86 (14.18) | 0-47 | 78.57 | 14.71 (29.18) | 0-91 | 78.57 |
| PH inpatient  Nights | 0.21 (0.96) | 0-5 | 7.14 | 0.36 (1.34) | 0-5 | 7.14 | 0.07 (0.27) | 0-1 | 7.14 |
| MH Day case  Contacts | 1.07 (2.97) | 0-15 | 28.57 | 2.07 (4.01) | 0-15 | 50.00 | 0.07 (0.27) | 0-1 | 7.14 |
| PH Day case  Contacts | 0.22 (0.80) | 0-4 | 11.11 | 0.07 (0.27) | 0-1 | 7.14 | 0.38 (1.12) | 0-4 | 15.38 |
| MH Outpatient  Contacts | 0.68 (1.25) | 0-5 | 32.14 | 0.50 (0.94) | 0-3 | 28.57 | 0.86 (1.51) | 0-5 | 35.71 |
| PH Outpatient  Contacts | 0.74 (2.09) | 0-10 | 22.22 | 0.79 (2.67) | 0-10 | 14.29 | 0.69 (1.32) | 0-4 | 30.77 |
| A&E  Attendances | 1.46 (1.62) | 0-8 | 78.57 | 1.50 (1.22) | 0-4 | 78.57 | 1.43 (1.99) | 0-8 | 78.57 |
| Ambulance  services | 0.19 (0.49) | 0-2 | 15.38 | 0.14 (0.53) | 0-2 | 7.14 | 0.25 (0.45) | 0-1 | 25.00 |
| **Community health and social care contacts** | | | | | | | | | |
| GP | 0.93 (1.44) | 0-5 | 35.71 | 0.86 (1.61) | 0-5 | 28.57 | 1.00 (1.30) | 0-3 | 42.86 |
| Practice nurse | 0.39 (0.92) | 0-4 | 21.43 | 0.36 (1.08) | 0-4 | 14.29 | 0.43 (0.76) | 0-2 | 28.57 |
| Other nurse | 2.25 (7.10) | 0-36 | 32.14 | 1.00 (1.88) | 0-7 | 42.86 | 3.50 (9.88) | 0-36 | 21.43 |
| CAMHS worker | 2.71 (3.15) | 0-11 | 71.43 | 3.36 (3.46) | 0-11 | 85.71 | 2.07 (2.79) | 0-10 | 57.14 |
| Psychiatrist | 0.96 (1.97) | 0-8 | 35.71 | 1.29 (2.49) | 0-8 | 42.86 | 0.64 (1.28) | 0-4 | 28.57 |
| CRT or HTT | 0.68 (1.91) | 0-9 | 25.00 | 0.57 (1.34) | 0-5 | 28.57 | 0.79 (2.39) | 0-9 | 21.43 |
| Talking therapy | 1.25 (3.22) | 0-12 | 25.00 | 1.50 (3.35) | 0-12 | 28.57 | 1.00 (3.19) | 0-12 | 21.43 |

**Table S6. ICCS and TAU-Related Service Use Over 6 Months**

|  | **All (n=28)** | | | **ICCS (n=14)** | | | **TAU (n=14)** | | |
| --- | --- | --- | --- | --- | --- | --- | --- | --- | --- |
| **Service** | **Mean (SD)** | **Range** | **% using** | **Mean (SD)** | **Range** | **% using** | **Mean (SD)** | **Range** | **% using** |
| **ICCS-specific worker** | | | | | | | | | |
| Psychiatrist | 1.39 (2.50) | 0-10 | 35.71 | 2.29 (3.17) | 0-10 | 50.00 | 0.50 (1.09) | 0-3 | 21.43 |
| Qualified CAMHS | 6.61 (7.38) | 0-22 | 60.71 | 11.64 (6.13) | 1-22 | 100.00 | 1.57 (4.57) | 0-17 | 21.43 |
| Unqualified CAMHS | 0.32 (1.02) | 0-5 | 14.29 | 0.64 (1.39) | 0-5 | 28.57 | 0.00 (0.00) | n/a | 0.00 |
| **Non-ICCS-specific worker** | | | | | | | | | |
| Psychiatrist | 1.07 (2.79) | 0-13 | 25.00 | 0.21 (0.58) | 0-2 | 14.29 | 1.93 (3.77) | 0-13 | 35.71 |
| Qualified CAMHS | 5.04 (6.22) | 0-30 | 82.14 | 6.29 (8.12) | 0-30 | 78.57 | 3.79 (3.33) | 0-10 | 85.71 |
| Unqualified CAMHS | 0.25 (1.32) | 0-7 | 3.57 | 0.00 (0.00) | n/a | 0.00 | 0.50 (1.87) | 0-7 | 7.14 |
| Full Day  Patient Ward | 2.00 (7.83) | 0-38 | 7.14 | 2.71 (10.16) | 0-38 | 7.14 | 1.29 (4.81) | 0-18 | 7.14 |
| Half Day  Patient Ward | 0.21 (1.13) | 0-6 | 3.57 | 0.43 (1.60) | 0-6 | 7.14 | 0.00 (0.00) | n/a | 0.00 |
| Psychiatric Inpatient Nights (PIN) | 9.14 (28.58) | 0-113 | 10.71 | 3.21 (12.03) | 0-45 | 7.14 | 15.07 (38.42) | 0-113 | 14.29 |
| Total Contacts  (excluding PIN) | 16.89 (16.67) | 0-82 | 92.86 | 24.21 (18.99) | 0-82 | 100.00 | 9.57 (10.06) | 0-38 | 85.71 |

**Table S7. Service Utilization Between ICCS and TAU Groups Over 6 Months**

|  | **All (n=28)** | | | **ICCS (n=14)** | | | **TAU (n=14)** | | | |
| --- | --- | --- | --- | --- | --- | --- | --- | --- | --- | --- |
| **Service** | **Mean (SD)** | **Range** | **% using** | **Mean (SD)** | **Range** | **%**  **using** | | **Mean (SD)** | **Range** | **%**  **using** |
| **Hospital** | | | | | | | | | | |
| PH Inpatient  Nights | 0.11 (0.42) | 0-2 | 7.14 | 0.21 (0.58) | 0-2 | 14.29 | | 0.00 (0.00) | n/a | 0.00 |
| PH Day case  Contacts | 0.14 (0.76) | 0-4 | 3.57 | 0.29 (1.07) | 0-4 | 7.14 | | 0.00 (0.00) | n/a | 0.00 |
| PH Outpatient  Contacts | 1.11 (2.87) | 0-12 | 32.14 | 1.14 (3.18) | 0-12 | 28.57 | | 1.07 (2.64) | 0-10 | 35.71 |
| A&E  Attendances | 0.54 (0.92) | 0-3 | 32.14 | 0.57 (0.94) | 0-3 | 35.71 | | 0.50 (0.94) | 0-3 | 28.57 |
| Ambulance  Services | 0.07 (0.38) | 0-2 | 3.57 | 0.00 (0.00) | n/a | 0.00 | | 0.14 (0.53) | 0-2 | 7.14 |
| Health-based  Place of safety | 0.04 (0.19) | 0-1 | 3.57 | 0.00 (0.00) | n/a | 0.00 | | 0.07 (0.27) | 0-1 | 7.14 |
| **Community health and social care contacts** | | | | | | | | | | |
| GP | 1.54 (1.73) | 0-8 | 71.43 | 1.29 (1.44) | 0-5 | 64.29 | | 1.79 (2.01) | 0-8 | 78.57 |
| Practice nurse | 0.29 (0.60) | 0-2 | 21.43 | 0.21 (0.43) | 0-1 | 21.43 | | 0.36 (0.74) | 0-2 | 21.43 |
| Other nurse | 1.25 (4.84) | 0-24 | 10.71 | 0.71 (2.67) | 0-10 | 7.14 | | 1.79 (6.40) | 0-24 | 14.29 |
| Talking therapy | 3.54 (5.73) | 0-24 | 46.43 | 2.29 (3.73) | 0-10 | 42.86 | | 4.79 (7.13) | 0-24 | 50.00 |
| Education MH Practitioner | 1.04 (2.74) | 0-12 | 21.43 | 1.07 (2.30) | 0-8 | 28.57 | | 1.00 (3.21) | 0-12 | 14.29 |
| Community Paediatrician | 0.14 (0.52) | 0-2 | 7.14 | 0.14 (0.53) | 0-2 | 7.14 | | 0.14 (0.53) | 0-2 | 7.14 |
| SEN  Coordinator | 0.29 (1.05) | 0-4 | 7.14 | 0.29 (1.07) | 0-4 | 7.14 | | 0.29 (1.07) | 0-4 | 7.14 |
| Social worker | 0.93 (2.67) | 0-13 | 21.43 | 0.64 (1.65) | 0-5 | 14.29 | | 1.21 (3.45) | 0-13 | 28.57 |
| OT | 0.29 (1.08) | 0-5 | 7.14 | 0.00 (0.00) | n/a | 0.00 | | 0.57 (1.50) | 0-5 | 14.29 |
| SLT | 0.04 (0.19) | 0-1 | 3.57 | 0.07 (0.27) | 0-1 | 7.14 | | 0.00 (0.00) | n/a | 0.00 |
| Drug/alcohol  Support worker | 0.14 (0.76) | 0-4 | 3.57 | 0.00 (0.00) | n/a | 0.00 | | 0.29 (1.07) | 0-4 | 7.14 |
| Helpline/advice  Service | 0.33 (0.78) | 0-3 | 18.52 | 0.46 (0.97) | 0-3 | 23.08 | | 0.21 (0.58) | 0-2 | 14.29 |

Abbreviations: MH Mental Health; PH Physical Health; A&E Accident and Emergency; GP General Practitioner; SEN Special Education Needs; OT Occupational Therapist; SLT Speech and Language Therapist.
